# Dynamics of charged particles in a solenoid and the accurate determination of the magnetic axis of a solenoid using measured field data

**DOI:** 10.1038/s41598-024-58645-z

**Published:** 2024-04-29

**Authors:** Gautam Sinha, Ritesh Malik

**Affiliations:** MDCL, RRCAT, Indore, MP 452013 India

**Keywords:** Energy science and technology, Engineering, Mathematics and computing

## Abstract

A fundamental understanding of the theory and measurement procedures for characterizing a solenoid is crucial for making a project cost-effective and achieving the desired performance for its use in a LINAC. This paper attempts to comprehend the beam dynamics of charged particles by considering the conservation of canonical angular momentum, which can take on positive, negative, and zero values. The analytical expression to generate a parallel beam using a solenoid is derived. The effect of the final beam size on the values of a solenoid’s spherical aberration coefficient and the position of the minimum beam size with respect to the focal length is studied using electron trajectories. A practical procedure to tune the above two parameters of a solenoid is demonstrated by simply modifying the demountable disks connected at each end. A mathematical model is proposed to accurately determine the axis of a solenoid and the magnetic centre using the measured magnetic field data, which is validated experimentally. Several solenoids, used in the 9.5 MeV 10 kW electron LINAC suitable for sterilizing medical products and food irradiation, have been characterised using this procedure.

## Introduction

Solenoids are widely used in applications ranging from undergraduate laboratory apparatus to high-tech sophisticated instruments like accelerators, electron microscopes and MRI. Several textbooks are available for the design and beam dynamics of solenoids^1–5^. Solenoids are commonly used for focusing low energy charged particles beams. Comparative studies of the focusing properties between the solenoid and quadrupole magnets are discussed in the literature^6^. However, FANL used a superconducting solenoid for focusing proton LINAC up to an energy of 100 MeV^7^. Apart from particle accelerators, superconducting solenoids are also used for MRI magnets^8,9^. For the SINBAD facility at DESY solenoids for beam focusing were employed, and the limit of their misalignment was studied. Beam experiences additional kick due to the mis-alignment of a solenoid, causing emittance growth. Therefore, alignment of the solenoid within 100 µm is demanded for the project^10^. Solenoids are also used in high power gun for RF sources^11^. The Axisymmetric magnetic field produced by a solenoid increases the electron beam transport by confining the beam and making it harder for its suitable application in microwave amplification. Focusing of charged particles beams by solenoids depends on various beam parameters like emittance growth due to spherical aberrations and non-linear space charge forces^12^. Solenoids are also used to improve the imaging performance of time-resolved electron microscopes^13^, as well as in particle therapy, radiographic diagnostics machines, and for rare isotope beams production^14^. An analytical expression for charged particle dynamics in a solenoid is derived using the conservation of canonical angular momentum^5,15^. All such calculations consider rotational symmetry of the magnetic field. Therefore, the alignment of the solenoid plays a significant role in achieving the desired performances^16–18^.

We have developed 9.5 MeV 10 kW electron LINACs in which large number of solenoids have been used. Despite having a large volume of literature, several factors still need to be discovered about the beam dynamics and characterization of the solenoids. This has motivated us to address these issues in this paper. We describe a viable solution with validated results using the experimental data and simulation results. To enhance the project's cost-effectiveness and ensure timely execution, a comprehensive understanding of the theory and the proper analysis of the experimental data are essential. The paper addresses issues related to two types of solenoids: (i) air core coil and (ii) coil surrounded by an iron jacket.

The following topics are discussed:

1. The derivation of the beam dynamics of charged particles by considering the conservation of canonical angular momentum with non-zero values involve a more detailed analysis of the particle motion than typically presented in available literatures^5,15^ that often assume only zero canonical angular momentum.
2. Comparison of the field quality of air core and coil surrounded by iron jacket solenoids.
3. The derivation of an approximate analytical formula to find the central field of a solenoid with an iron jacket.
4. Utilizing a solenoid for the generation of parallel beams.
5. The procedure involves tuning the values of the spherical aberration coefficient and focal length of a solenoid, both of which significantly impact its optical properties. This is achieved by modifying the demountable disks connected at each end.
6. Determination of the magnetic axis and centre of a solenoid from the measured magnetic field data.

Some of the results are verified using single particle trajectory using Opera Simulation Software^19^. The self-Field produced by the beam is ignored in the calculation.

The axis of a solenoid can be determined by means of the single stretched wire method^20^. Electrical voltage is induced across a wire when its ends move in the opposite direction with respect to the centre of the solenoid. The induced voltage is at a minimum when the wire coincides with the solenoid axis. However, it is important to note that this method provides an integrated measurement and is not suitable for detecting the error field profile of a solenoid. The accuracy is lower because the transverse field intercepts only near the edges of the solenoid.

The magnetic axis of a solenoid was determined by moving two sets of induction coils along the longitudinal direction inside the solenoid^21^. Induced voltage was measured on the fly, and the flux density was expressed as a Taylor series. The magnetic axis was identified by detecting the minimum of the local field gradient. However, a limitation of this procedure is the need for a 2.5 m travel range of the linear movement system to characterize a 0.390 m long solenoid^21^. Additionally, achieving an accuracy of 50 µm requires multiple runs (e.g., 6 runs) per direction for averaging. Furthermore, the minimum of the local gradient is not sharp, as observed in the measured data, and the field calculated by this method is averaged over the area of the search coil. Therefore, this method does not provide a point measurement.

In contrast to the previous method, we propose a new procedure for axis determination using a 3D Hall sensor with a magnetic field sensitive volume of (0.1 × 0.01 × 0.1) mm^3^ and a magnetic field accuracy of 100 PPM, which are readily available in the market. A linear travel range exceeding the length of the solenoid by 10% is sufficient for accurate axis determination. The zero-crossing method is employed for this purpose, and periodic calibration of the Hall sensor is conducted using an NMR probe. Magnetic field data is acquired at a 1.0 k-sample/s sampling rate for rapid averaging of measured field data. These features render the proposed method accurate to better than 50 µm, robust, and fast. Additionally, this process minimizes error transverse fields of the solenoid, which cause emittance growth, to within 1 G. The measurement of error fields enables the estimation of mechanical inaccuracies present in the solenoid, providing opportunities for further improvement.

Solenoids play a crucial role in halo removal for intense high-energy beams in storage rings and colliders^22^. Projects such as the Hollow Electron Lens^23,24^ and Electron Cooler^25^ require the precise alignment of solenoids within 50 µm. Our proposed measurement method fulfills this stringent requirement.

## Beam dynamics in a magnet having rotational symmetry like solenoid

Consider a particle of charge q and momentum, P is moving in Z direction through a solenoid. Z is the symmetric axis of the solenoid. The momentum P can be expressed as $\overrightarrow {P} = \overrightarrow {P}_{Z} + \overrightarrow {P}_{ \bot }$. Assume that $\overrightarrow {P}_{Z} \gg \overrightarrow {P}_{ \bot }$. Magnetic field in a solenoid has rotational symmetry. Therefore, the canonical angular momentum is conserved. The magnetic field of a solenoid can be expressed as^4,21,26^

1 $$\begin{aligned} \overrightarrow {B(r,Z)} & = B_{r} (r,Z)\hat{r} + B_{Z} (r,Z)\hat{z} \hfill \\ B_{r} (r,Z) & = - \frac{{B_{Z0}^{\prime} (Z)r}}{2} + \frac{{B_{Z0}^{\prime\prime\prime} (Z)}}{16}r^{3} - ......... \, \hfill \\ B_{Z} & = B_{Z0} (Z) - \frac{{B_{Z0}^{\prime\prime} (Z)}}{4}r^{2} + ......... \, \hfill \\ \end{aligned}$$

where *B*_*z*0_(*Z*) = on-axis field and $\hat{r}$ and $\hat{z}$ are the unit vector along r and Z direction, respectively.

In this paper we will consider the linear term only.

Canonical angular momentum

2 $$\begin{aligned} \ell &= [\overrightarrow {r} \times (\overrightarrow {P} + q \cdot \overrightarrow {A} )]\hat{z} = {\text{constant}} \\ \overrightarrow {B} & = \overrightarrow {\nabla } \times \overrightarrow {A} \end{aligned}$$

where *A* is vector potential.

Equation (2) can have three possible solutions depending on the value of the constant is

1. Positive
2. Negative
3. Zero

The possible solutions are examined below.

Magnetic field, $\overrightarrow {B}_{Z}$ of a solenoid can have vector potential

3 $$A_{\theta } = \frac{{rB_{Z} }}{2},{\text{ using }}[2\pi r \cdot A_{\theta } = \pi r^{2} B_{Z} ]$$

We know that the trajectory of a charge particle q with an average momentum in Z direction inside a solenoid, is a helix. We have solved this under impulsive approximation^5,15^, which means the particle experiences azimuthal kick only when it passes through the fringe field regions of the solenoid. Therefore,

4 $$\begin{aligned} P_{Z,out} & = P_{Z,in} \hfill \\ P_{\theta ,out} & = P_{\theta ,in} + \frac{qBr}{2} \hfill \\ P_{r,out} & = P_{r,in} \hfill \\ \end{aligned}$$

Let the distance of the centre of the helix from the magnetic axis be $R_{d}$. The radius of the helix,$R_{h}$ can be obtained from the force equation

5 $$\begin{aligned} \overrightarrow {F} & = \gamma m\overrightarrow {a} \, \hfill \\ {\text{or, }}\frac{{\gamma mV_{ \bot }^{2} }}{{R_{h} }} & = qV_{ \bot } B_{Z} \hfill \\ or, \, R_{h} & = \frac{{\gamma mV_{ \bot } }}{{qB_{Z} }}{ = }\frac{{P_{ \bot } }}{{qB_{Z} }} \hfill \\ \end{aligned}$$

From Eqs. (2), (3) and (5) we have

6 $$\begin{aligned} \ell_{Z} & = (R_{d} - R_{h} )P_{ \bot } + q\vec{r} \times \left( {\frac{{rB_{Z} }}{2}} \right)\hat{\theta } \, \hfill \\ & = (R_{d} - R_{h} )(R_{h} \, q \, B_{Z} ) + \frac{{qB_{Z} }}{2}(R_{d} - R_{h} )^{2} \, \hfill \\ & = \frac{{qB_{Z} }}{2}\left( {R_{d}^{2} - R_{h}^{2} } \right) \hfill \\ \end{aligned}$$

**Figure 1:**
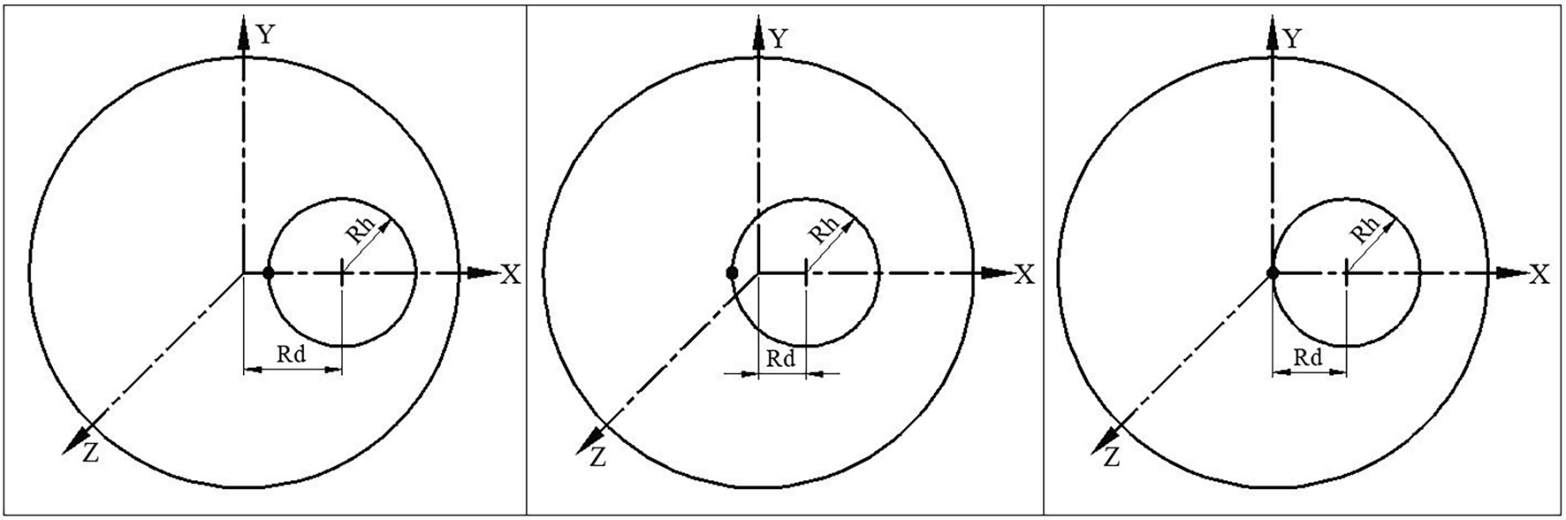
Case 1: In this case the trajectory does not include the magnet axis, O ($R_{d} > R_{h}$). Case 2: In this case the trajectory includes the magnet axis, O ($R_{d} < R_{h}$). Case 3: In this case the trajectory passes through the magnet axis, O ($R_{d} = R_{h}$).
Case 2: For $R_{d} < R_{h}$,$\ell_{Z}$ is negative. Hence, the trajectory includes the magnet axis as shown in Fig. 1.
Case 3: For $R_{d} = R_{h}$, $\ell_{Z}$ is zero. Hence, the trajectory passes through the magnet axis as shown in Fig. 1. In this case when the particle is launched at a distance $2R_{h}$ from the centre of the magnetic axis, then the radius of the helix will be $R_{h}$, as documented in the literature $\left( {R_{h} = \frac{R}{2}} \right)$^15^.

Case 1: For $R_{d} > R_{h}$,$\ell_{Z}$ is positive. Hence, the trajectory does not include the magnet axis as shown in Fig. 1.

Graphical representation of Eq. (6) is shown in Fig. 1 for all the three cases.

**Figure 2:**
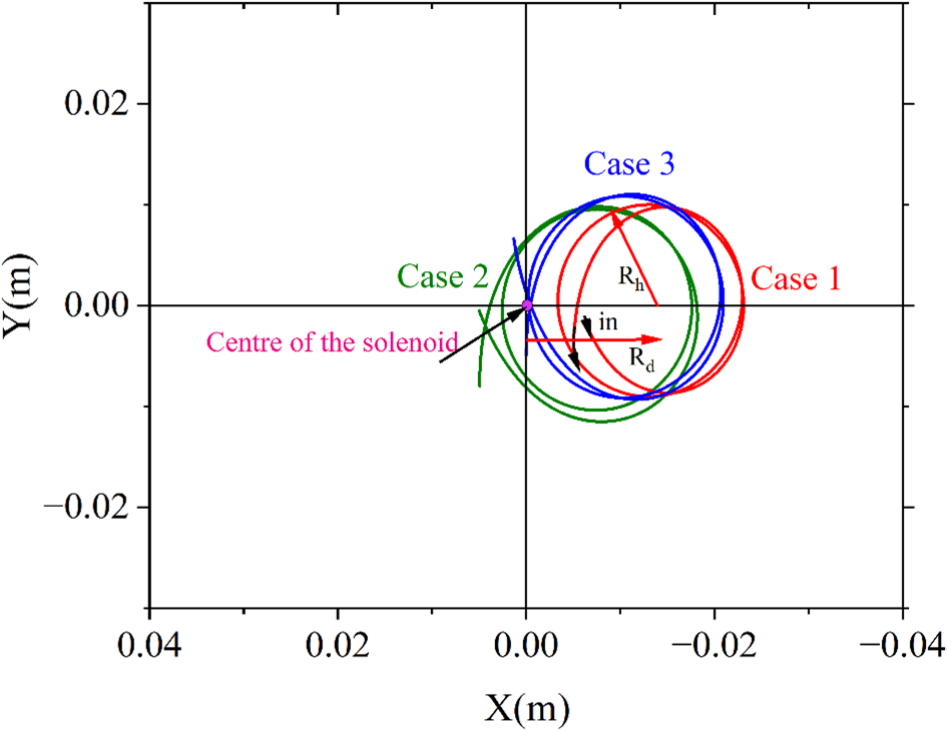
X–Y projection of the trajectory of a 9 MeV electron through a solenoid with a peak magnetic field of 0.1192T. Electron is projected off-axis with a small angle with Z-axis to have a small momentum in the perpendicular direction for both case 1 and case 2.

Particle trajectory of a 9 MeV electron through a 0.384 m long solenoid with a peak magnetic field of 0.1192 T is shown in Fig. 2. The actual trajectory shows a very similar nature to that predicted by Eq. (6). Case 1 and case 2 are closer to practical scenarios because the beam always has finite emittance. Self-field produced by the beam is ignored in the calculation.

## Use of solenoid to generate parallel beam and focusing

A particle of charge q and momentum P is launched at a small angle $\alpha_{1}$ with respect to the Z axis from a point (0, y_1_, − Z_1_) that reaches at the solenoid entrance at P_1_(α_1_Z_1_, y_1_,0) with momentum (P_in_, 0, P) where P_in_ = P $\alpha_{1}$. The axis of the solenoid is in the Z direction with $B = B_{Z} \hat{Z}$ extending from Z = 0 to L.

At the entrance of the solenoid, the fringe field provides an azimuthal kick to the charge particle, which is represented by $\vec{P}_{\theta 1} = - \frac{{qBR_{in} }}{2}$. Therefore,

7 $$P_{nori} = \sqrt {P_{\theta 1}^{2} + P_{in}^{2} } = \frac{{qBR_{in} }}{2}\sqrt {1 + \left( {\frac{2P}{{qBZ_{1} }}} \right)^{2} } = qBR_{h}$$

From the triangle ΔOP_1_P_2_,

8 $$\theta = \frac{{\theta_{h} }}{2}$$

Because of the azimuthal kick at the entry of the solenoid, the particle rotates by an azimuthal angle $\theta_{h}$ as it traverses along the length of a solenoid of length L.

9 $$\theta_{h} = \frac{{V_{nori} t}}{{R_{h} }} = \frac{{V_{nori} }}{{R_{h} }} \cdot \frac{L}{{V_{Z} }} = \frac{{P_{nori} \cdot L}}{{P \cdot R_{h} }} = \frac{qBL}{P}$$

We have by using Eqs. (7) and (8)

10 $$\theta = \theta_{1} + \theta_{2} = \frac{qBL}{{2P}}$$

Just before exit of the solenoid the particle will have both radial $\left( {P_{out} } \right)$ and azimuthal $\left( {P_{\theta 2} } \right)$ momentum that can be expressed as

11 $$\begin{aligned} P_{out} & = - P_{noro} \sin \theta_{2} = - P_{noro} \frac{{\sin \theta_{2} }}{{\cos \theta_{2} }}\cos \theta_{2} = - P_{noro} \tan \theta_{2} \cos \theta_{2} \hfill \\ & = - P_{noro} \tan \theta_{2} \frac{{R_{out} }}{{2R_{h} }} \hfill \\ \end{aligned}$$

From triangle ΔOA_h_P_2_, we have $R_{out} = 2R_{h} \cos \theta_{2}$.

12 $$P_{\theta 2} = - P_{noro} \cos \theta_{2} = - \frac{{qBR_{out} }}{2}$$

However, at the exit, the fringe field will provide a kick in the azimuthal direction, incrementing the azimuthal momentum by an amount $\frac{{qBR_{out} }}{2}$. Therefore, the resultant azimuthal momentum will be zero, which will make the canonical momentum zero.

So, after exiting the solenoid, the particle will have transverse momentum only in the radial direction. This will guide the charge particle towards the solenoid axis. Assume that at a distance $Z_{2}$ from the end of the solenoid, the particle will cross the solenoid axis, making an angle $\alpha_{2}$ with the Z axis.

13 $$\begin{aligned} \frac{{R_{out} }}{{Z_{2} }} & = \alpha_{2} = \frac{{P_{out} }}{P} \hfill \\ or, \;\; Z_{2} & = \frac{{R_{out} P}}{{P_{out} }} = \frac{{2R_{h} P}}{{P_{noro} \tan \theta_{2} }} = \frac{{2R_{h} P}}{{qBR_{h} \tan \theta_{2} }} = \frac{2P}{{qB\tan \theta_{2} }} \hfill \\ \end{aligned}$$

If the particle is launched on the axis and at the entry of the solenoid, then $Z_{1} = 0 \, and \, R_{in} = 0.$

**Figure 3:**
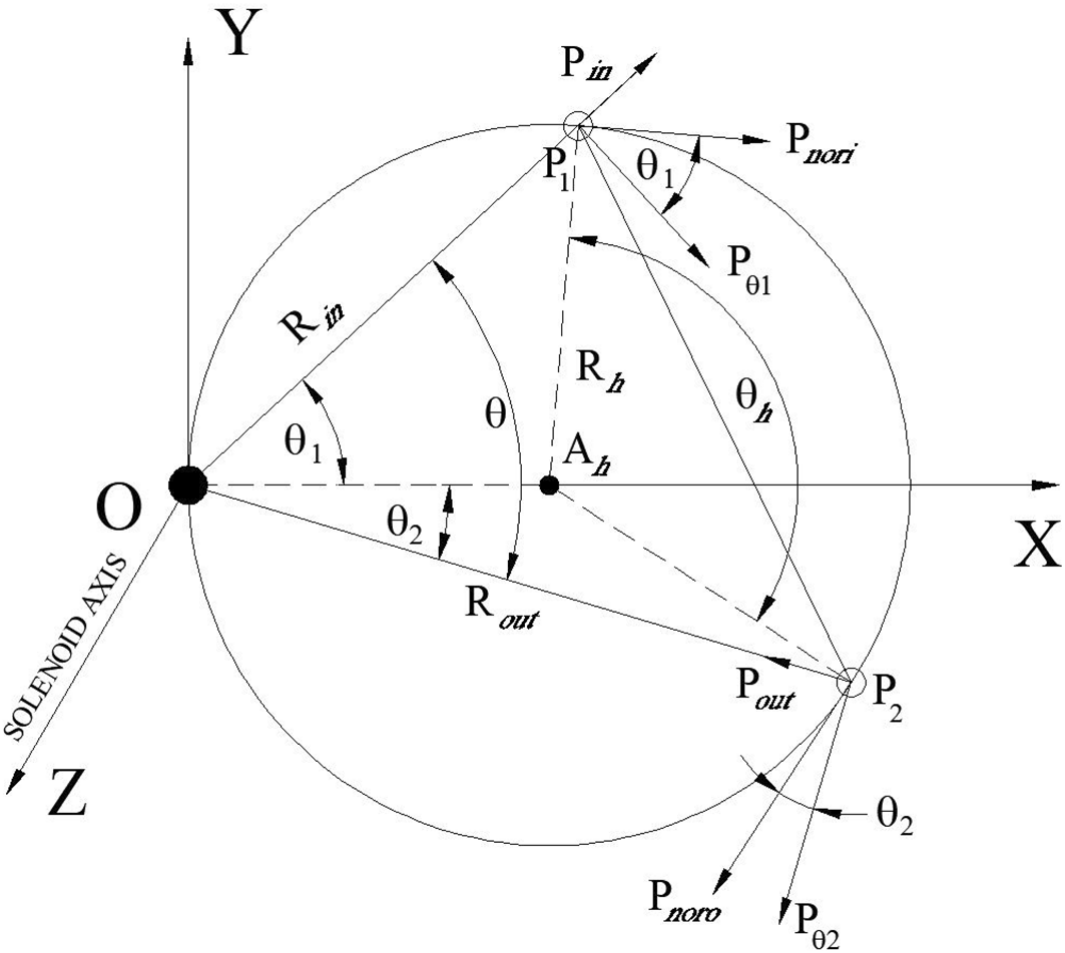
Projection of trajectory on X–Y plane at the centre of the solenoid of length L(Z). O and A_h_ are the axis of the solenoid and helical trajectory, respectively. Particles enter at point P_1_(R_in_, θ_1_,0) and exit of the solenoid at P_2_ (R_out_, − θ_2_, L).

In that case, we get from Fig. 3, $\theta_{1} = \pi /2$ and hence $\theta_{2} = \frac{qBL}{{2P}} - \frac{\pi }{2}$.

Equation (13) can be written as

14 $$Z_{2} = \frac{2P}{{qB\tan \left( {\frac{qBL}{{2P}} - \frac{\pi }{2}} \right)}} = \frac{2P}{{qB}}\tan \left( {\frac{qBL}{{2P}}} \right)$$

If $\tan \left( {\frac{qBL}{{2P}}} \right) = \infty {\text{ then }}Z_{2} \to \infty$, This is the condition of generating parallel beam.

So, $\frac{qBL}{{2P}} = (2n + 1)\frac{\pi }{2}$ where n is integer 1, 2, 3 …..etc.

$$for \, n = 0, \, L = \frac{P\pi }{{qB}}$$

15 $$P = \frac{q}{\pi }\int {B \cdot dl}$$

If a particle of momentum P and charge q is launched on the axis inside a solenoid which satisfies the Eq. (15), then a parallel beam will come out of the solenoid.

From Fig. 3, we have

16 $$\tan \theta_{1} = \frac{{P_{in} }}{{P_{\theta 1} }} = \frac{{P\alpha_{1} }}{{qBR_{in} /2}} = \frac{2P}{{qBZ{}_{1}}}$$

Using Eqs. (10) and (16) we get $\tan \theta_{2} = \tan \left( {\frac{qBL}{{2P}} - \tan^{ - 1} \frac{2P}{{qBZ_{1} }}} \right)$.

Using Eq. (13)

17 $$Z_{2} = \frac{2P}{{qB\tan \left( {\frac{qBL}{{2P}} - \tan^{ - 1} \frac{2P}{{qBZ_{1} }}} \right)}}{ = }\frac{2P}{{qB}}\frac{{1 + \frac{2P}{{qBZ_{1} }}\tan \frac{qBL}{{2P}}}}{{\tan \left( {\frac{qBL}{{2P}}} \right) - \frac{2P}{{qBZ_{1} }}}}$$

If $$Z_{1} \to \infty$$ then

18 $$Z_{2} = f = {\text{focal length = }}\frac{2P}{{qB\tan \left( {\frac{qBL}{{2P}}} \right)}}$$

For small lens approximation

19 $$f = \frac{{4P^{2} }}{{q^{2} \int {B^{2} \cdot dl} }}$$

If an electron with momentum P is launched on the axis inside the solenoid, at an angle, θ, with the solenoid axis and the field integral satisfies Eq. (15), then the electron will emerge parallel to the axis. For example, if the solenoid extends from − 0.2 to 0.2 m and has a field integral $\int\limits_{ - 0.15}^{\infty } {B_{Z} dZ} = 0.04499{\text{ Tm}}$ then the trajectory of an electron with an energy of 4.3262 MeV will become parallel to the solenoid axis. Similarly, by varying the injection point, electrons with different energies can be made parallel.

## Estimation of the spot size on the focal plane

Several factors control the beam size at the focal plane. However, major contributions come from (i) Beam emittance, (ii) Chromatic aberration due to the energy spread of the beam, and (iii) Spherical aberration arising from the field profile of the solenoid. The radius of the final beam $\left( {R_{f} } \right)$ can be correlated with the initial beam $\left( {R_{0} } \right)$ through the relation^4^

20 $$R_{f}^{2} = \left( {\frac{\varepsilon }{{R_{0} }}f} \right)^{2} + \left( {2R_{0} \frac{\Delta \gamma }{\gamma }} \right)^{2} + \left( {C_{s} R_{0}^{3} } \right)^{2}$$

where $$\varepsilon = {\text{ emittance}}, \, \frac{\Delta \gamma }{\gamma } = {\text{ energy spread and }}C_{s} {\text{ = spherical aberation coefficient}}{.}$$

21 $$C_{s} = \frac{1}{2}\frac{{\int {\left( {\frac{{dB_{z} }}{dz}} \right)^{2} dz} }}{{\int B_{Z}^{2} dz}}$$

**Figure 4:**
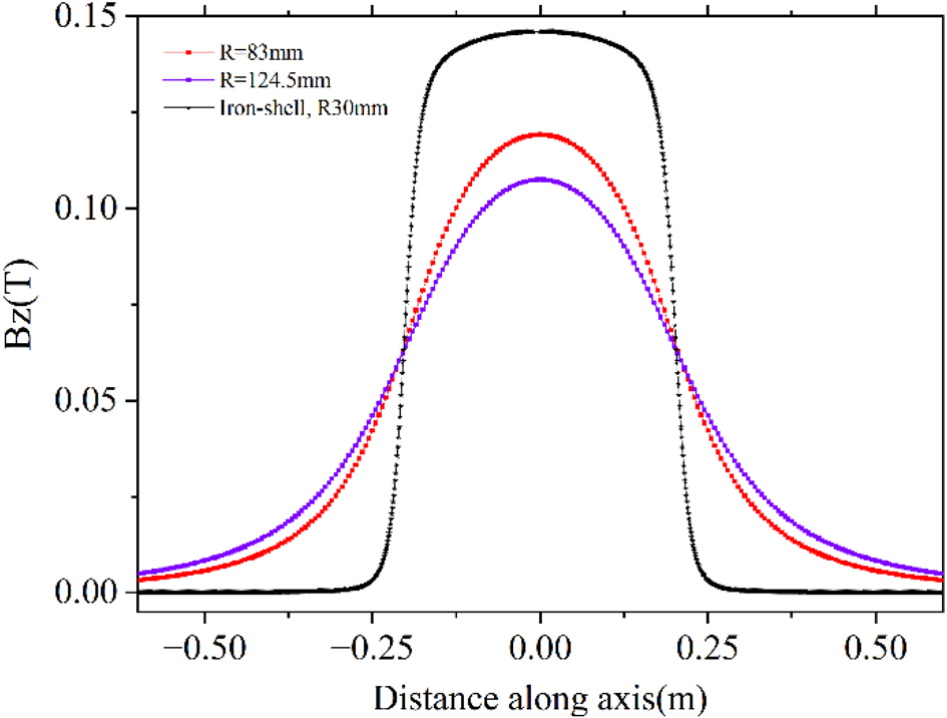
Field profile for three different cases for the same NI but different geometrical parameters of solenoids that produce same $\int {B_{Z} dZ}$.

However, in this article, we will only consider the effect of $C_{s}$^4^, which depends on the field profile of the solenoid by studying the single-particle trajectory. The motion of a charged particle inside a solenoid is coupled motion. The solenoid rotates and focus the beam, as explained in Eqs. (10) and (18). The angle of rotation depends on $\int {B_{Z} dZ}$ which is governed only by the total current, NI and not on the geometrical parameters of the solenoid. This can be obtained using Amper’s law, which is also observed in simulation and measurement. We have considered three cases (i) a coil of length, inner, and outer radii are 384 mm, 83 mm and 210 mm, respectively, (ii) the inner radius of the coil is increased by 1.5 times to 124.5 mm, and (iii) the coil used in case (i) is put inside a 12 mm thick iron jacket. The total length, inner and outer radii of the solenoid with iron jacket are 418 mm, 30 mm and 225 mm, respectively. In all three cases, the total current in the solenoid is kept as 45,800 Amp. turn. $\int {B_{Z} dZ}$ for all three cases is found to be 0.05753 Tm. Peak magnetic fields are 0.119251 T, 0.107529 T, and 0.146073 T, respectively. Field profiles for the three different cases are shown in Fig. 4. The value of $C_{s}$(1/m^2^) for the three different cases are 8.23, 6.44 and 35.84, respectively.

It is clear from Fig. 4 that iron jacket increases the peak field but reduces the fringe field region and increases $C_{s}$. In any accelerator, components are placed close to each other, and fringe fields cause interference between various components, which may not be desirable in most cases. Several magnetic elements are used in our 9.5 MeV 10 kW electron LINAC. We have experimentally observed the undesirable effect of fringe fields. Therefore, there shall be a trade-off between controlling the field span and the value of $C_{s}$ for a practical machine.

Field profile of a solenoid without iron can be calculated analytically. Peak field with iron jacket can be estimated using the relation $B = \frac{{\mu_{0} NI}}{gap} = \frac{{4\pi 10^{ - 7} \times 45800}}{0.394} = 0.146076T$, which is close to the simulated (and measured) value.

**Figure 5:**
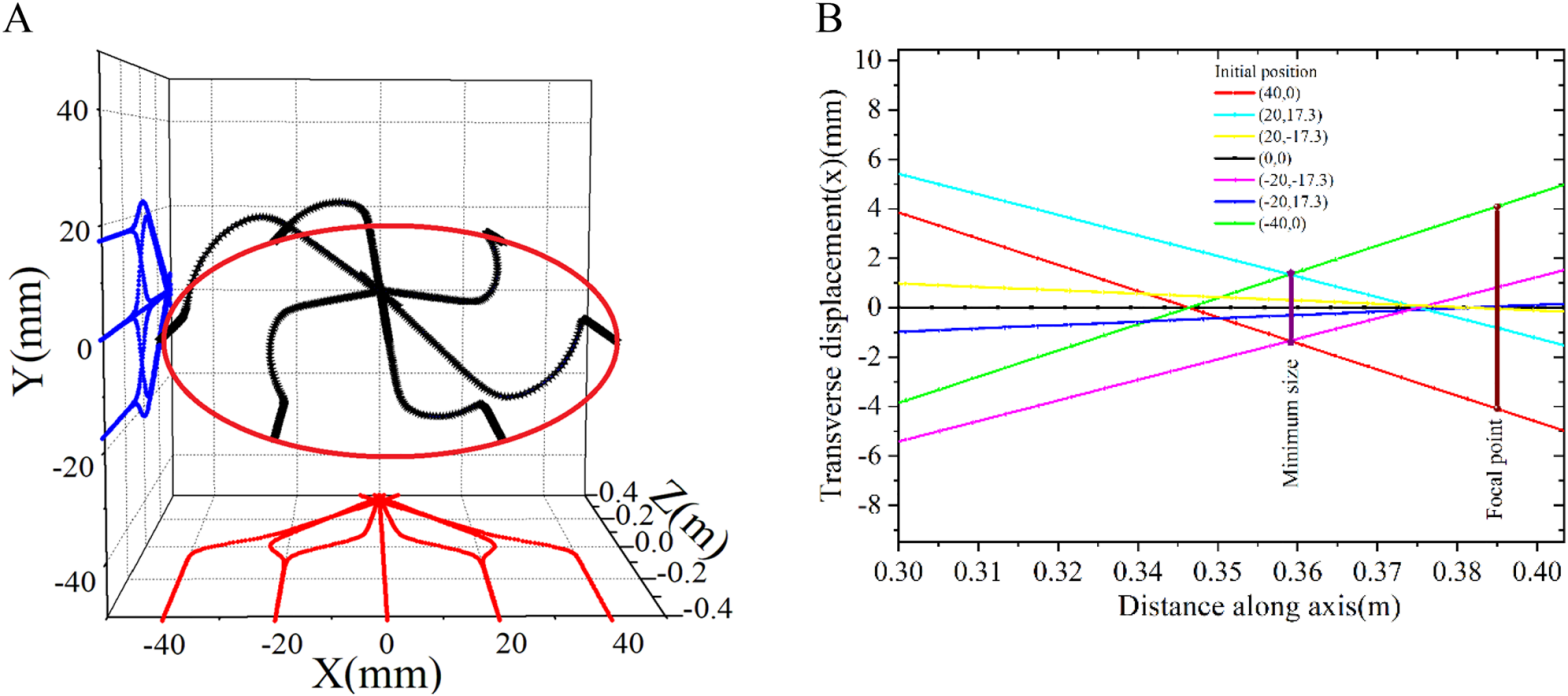
3-D trajectory of elliptical beam through a solenoid of focal length 0.39 m and its projection on the transverse planes. Initial beam size along X is ± 40 mm and final sizes are ± 1.35 mm and ± 4.08 mm at 0.359 mm and 0.390 mm, respectively (**A**). Trajectory is zoomed to show the size of the beam at focal point (**B**).

The theoretical prediction as given in Eq. (20) demands lower value of $C_{s}$ for better focusing. But how low, that needs to be estimated. We have done single particle trajectory to find the effect of $C_{s}$ on the final beam size. Particles at six different points on an ellipse are launched along the Z direction. 3-D trajectory and its projection on the transverse planes are shown in Fig. 5. Initial beam size along X is ± 40 mm and final sizes are ± 1.35 mm and ± 4.08 mm at 0.359 mm and 0.390mm, respectively. Therefore, it is clear that the beam size is nearly 3 times smaller at 0.359 m compared to the size at the focal plane for large beam size. It is important that the target be placed prior to the focal plane in order to obtain the minimum beam size.

Now, we consider two entirely different solenoids but having similar $C_{s}$ value as given in Table1.

**Table 1:** Various important parameters of two different solenoids.

	Sol-1	Sol-2
$\int\limits_{ - \infty }^{\infty } {B_{Z}^{2} } dZ{\text{ (T}}^{{2}} {\text{m)}}$	2.742E−02	1.748E−02
$\int\limits_{ - \infty }^{\infty } {\left( {\frac{{dB_{Z} }}{dZ}} \right)^{2} dZ} {\text{ (T}}^{{2}} {\text{/m)}}$	5.283	3.193
$C_{s} {\text{ (1/m}}^{{2}} {)}$	96.34	91.33
$\int\limits_{ - \infty }^{\infty } {B_{Z} dZ} {\text{ (Tm)}}$	7.237E−02	6.711E−02
B peak(T)	0.5171	0.3113
$L_{eff} (m) = \frac{{\int\limits_{ - \infty }^{\infty } {\left( {dB_{Z} dZ} \right)^{2} } }}{{\int\limits_{ - \infty }^{\infty } {B_{Z}^{2} dZ} }}$	0.191	0.257
Inner diameter(mm) of the iron jacket	100	50
Total length of the magnet(m)	0.320	0.225

**Figure 6:**
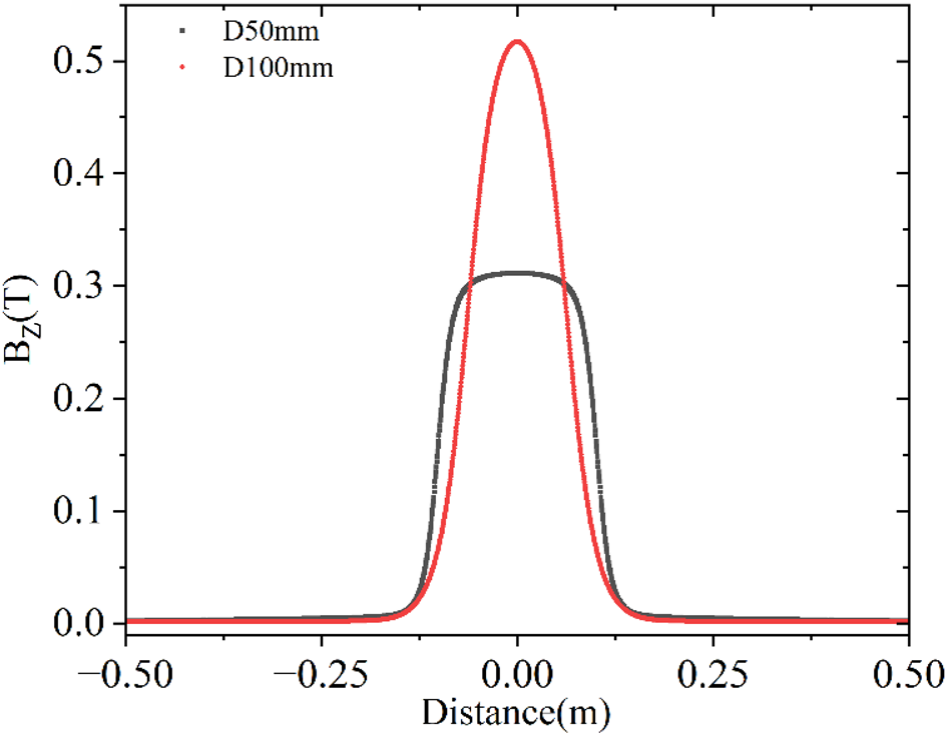
Field profile of the two different solenoids, Sol-1 (D100mm) and Sol-2 (D50mm) having similar $C_{s}$ value.

**Figure 7:**
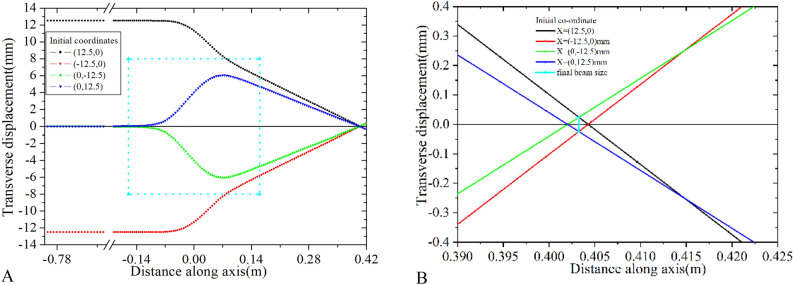
Trajectory of the particle through Sol-1 (D100mm). Rectangular area surrounded by dotted line represents the edges of the solenoid (A). Trajectory is zoomed to show the size of the focus beam (**B**).

Field profile of the two different solenoids, Sol-1 and Sol-2 is presented in Fig. 6. Important parameters of the two different solenoids are also shown in Table 1. An electron located at four different points of a 12.5 mm radius circle is launched one by one along the Z direction and its trajectories are shown in Fig. 7. According to the theory, the final beam size will be 0.188 mm at the focal plane. The focal distance, as calculated using Eq. (18) is around 0.419m, where the beam size is slightly larger than the theoretical prediction. However, the minimum beam size is considerably smaller at 0.40m which is 0.025mm, as observed in Fig. 7. Therefore, it is possible to reduce the beam size from 12.5 mm to 0.025 mm. Even in the case of such a high value of $C_{s}$, it is possible to reduce the beam size by nearly 500 times. However, the other parameters will affect the final beam size, as explained in Eq. (20). Therefore, there is no point in demanding a very low value of $C_{s}$ unless it is required. This kind of beam size reduction is acceptable for most LINACs including ours.

**Figure 8:**
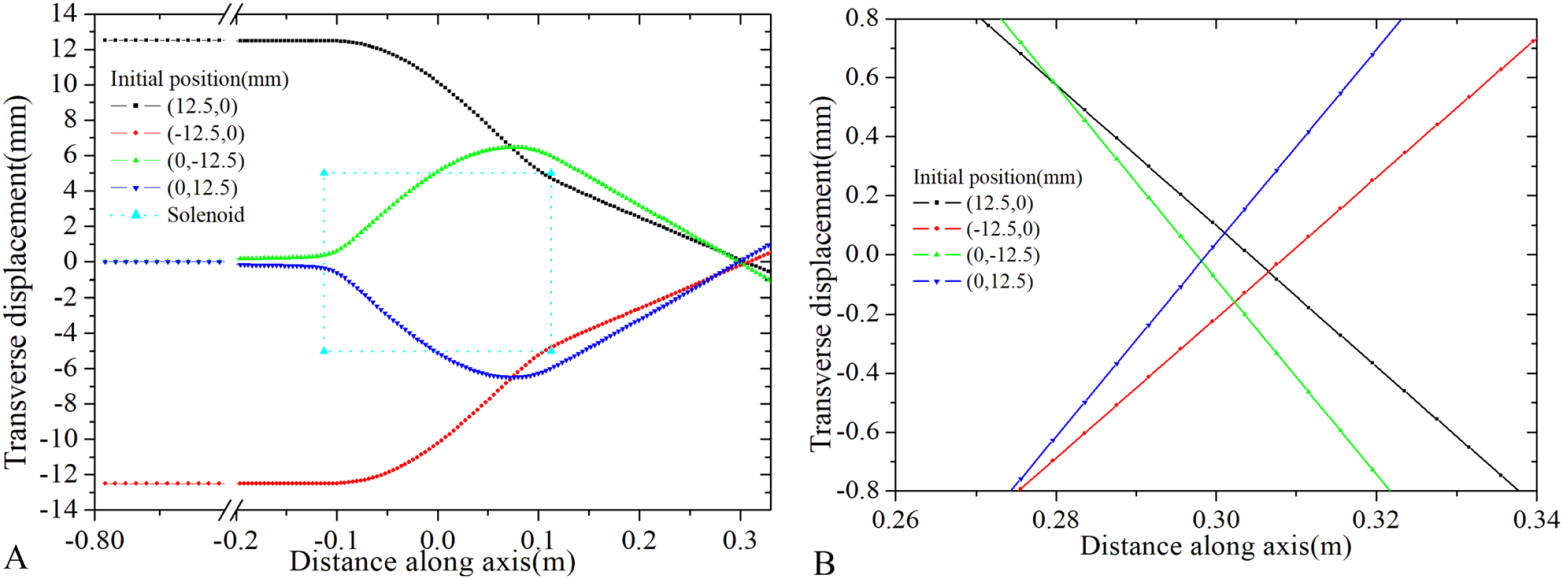
Trajectory of the particle through Sol-2 (D50mm). Rectangular dotted line represents the edges of the solenoid (**A**). Trajectory is zoomed to show the size of the focus beam at minimum beam position (**B**).

However, in case of Sol-2, degradation of beam quality is observed, where the ratio of the inner radius of the solenoid to the radius of the input beam $\left( {\frac{{R_{iSol} }}{{R_{0} }}} \right)$ is 2. Whereas, the ratio is 4 for Sol-1. Trajectory of the electron is shown in Fig. 8 for Sol-2.

In the case of Sol-2, despite having a similar $C_{s}$ value, the minimum beam size is around 0.2 mm. Therefore, not only the value $C_{s}$ plays a significant role, but the ratio $\left( {\frac{{R_{iSol} }}{{R_{0} }}} \right)$ also influences the reducing of bean size. Consequently, in most LINAC machines, the inner radius of the solenoid is kept large compared to the beam radius.

**Figure 9:**
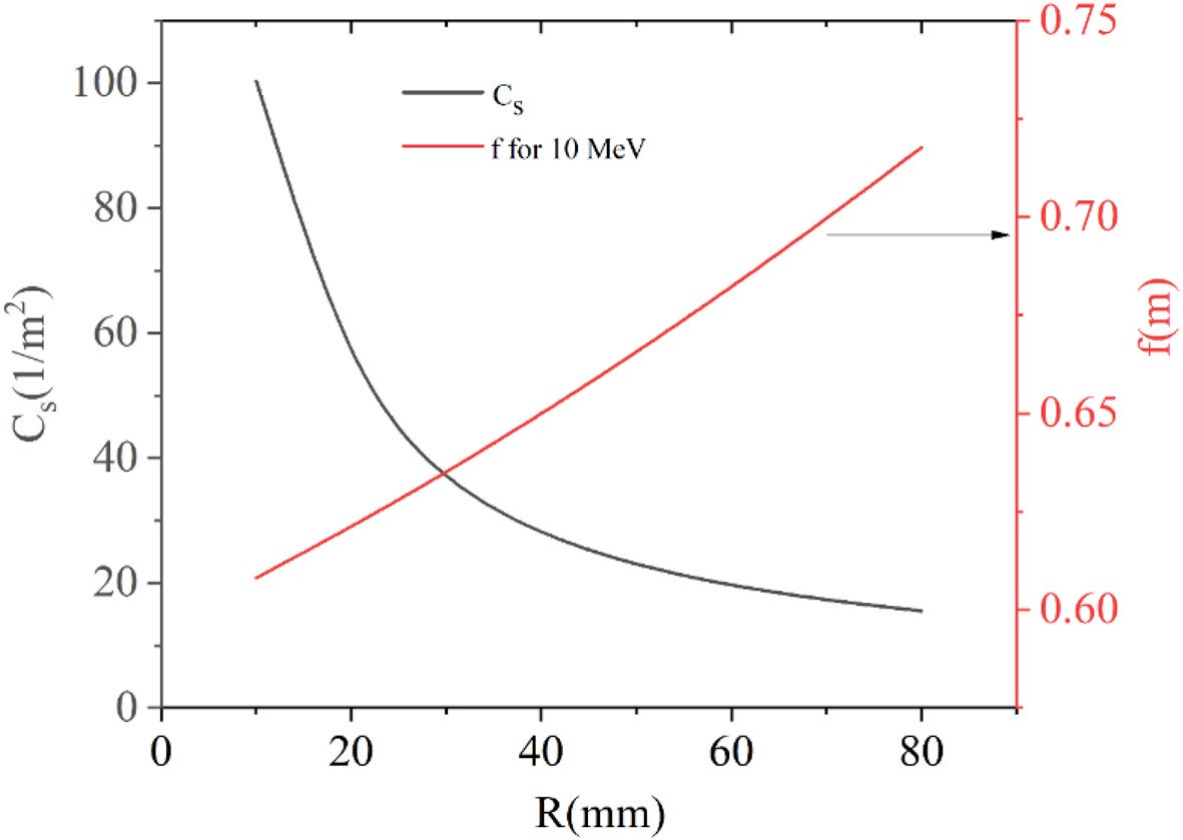
Variation of spherical aberration coefficient and focal length for 10 MeV electron with the radius of the inner iron jacket.

We have already shown that $\int {B_{Z} dZ}$ will depend only on the total current of the solenoid. However, various other important parameters of a solenoid such as $C_{s}$, focal length, and peak magnetic field, can be altered as needed by varying the inner radius of the iron jacket while keeping NI constant. As we decrease the inner radius, the peak central field and $C_{s}$ will increase, but the fringe field zone will reduce. At the same time, the focal length reduces, i.e., the focusing power increases. Therefore, based on the requirement of the project, several properties of a solenoid can be tuned by varying the inner radius of the iron jacket. A de-mountable disk at both the ends are provided on the iron jacket to make the solenoid versatile. Several such magnets are built, characterised, and used in various applications. Figure 9 shows the variation of spherical aberration coefficient and focal length for 10 MeV electrons with the radius of the inner iron jacket.

## Find the axis of a solenoid using measured magnetic field data

**Figure 10:**
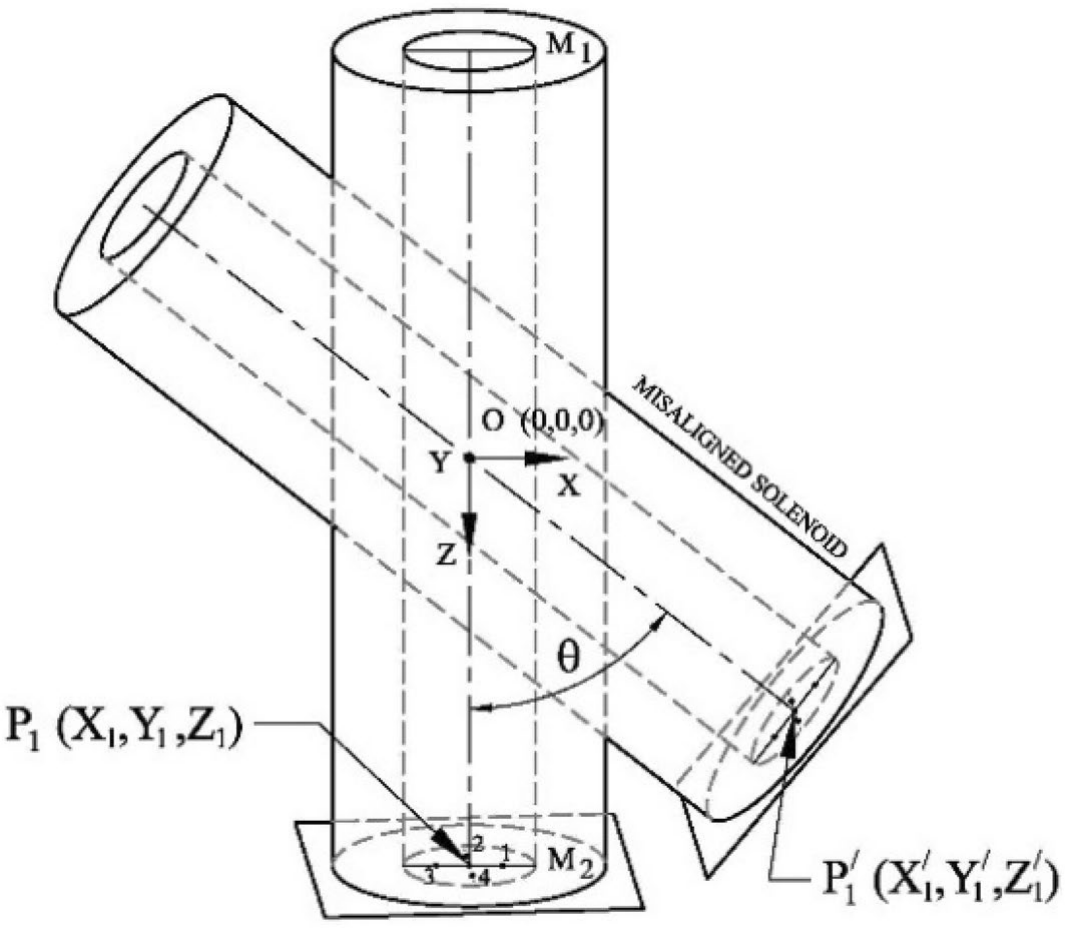
Frame of reference of 3D sensor positioning bench (X, Y, Z) and mis-aligned solenoid (X′, Y′, Z′).

Axially symmetric magnetic fields produced by a solenoid are utilized in numerous applications. Therefore, it is crucial to determine the axis of a solenoid to achieve the desired performance. Mathematical modeling and measurement procedures are described with practical examples. The field at the centre of an ideal solenoid is produced only along axial direction, B_Z._ Both the transverse field, B_X_ and B_Y_ are zero on the axis (0, 0, Z), as shown in Fig. 10. An integrated 3-axis Hall sensor is essential to detect this condition. On the Y = 0 plane, B_Y_ is zero, and B_X_ linearly varies with X, crossing 0 at the centre. For a given Z, B_X_(−X) = −B_X_(X) on the Y = 0 plane. B_X_ is maximum at the edges of the magnet; therefore, experiments are conducted at the edges to achieve better accuracy. The above properties are used to detect the centre of the solenoid using simulated and experimental data.

A 3-axis sensor positioning bench (PB) and a 3-axis SENIS Hall sensor attached to a 3MH5C tesla meter are used for field measurement. The magnetic field sensitive volume of the sensor is approximately (0.1 × 0.01 × 0.1) mm^3^, and its angular accuracy is ± 0.5°. To align the Hall sensor horizontally (in the X–Y plane), the sensor is moved inside an aligned dipole magnet as part of the initial alignment procedure. The solenoid is placed on the magnet alignment bench, which has a circular base. Both the PB (X,Y,Z) and the Hall sensor are positioned horizontally. B_Y_ is adjusted to zero at both ends from (− 0.03, 0, L/2) to (0.03, 0, L/2) M_1_ line and from (− 0.03, 0, − L/2) to (0.03, 0, − L/2) M_2_ line by using the alignment table. Here, L is the length of the solenoid. This alignment procedure is used to define the X–Y plane.

Suppose the solenoid is tilted by an angle θ about Y axis with respect to the reference frame of PB about the point O. The frame of reference of the mis-aligned solenoid is defined as X′Y′Z′ (Fig. 10). The field B_Z_ along the Z axis is measured. $\frac{{\partial B_{Z} }}{\partial Z}{\text{ vs}}{. }Z$ changes sign at Z = 0. This plot defines the magnetic centre along the length. Any lateral shift along the length is adjusted based on the measurement.

The following two matrices are used to convert the position and field from the PB frame (X, Y, Z) to the solenoid frame (X′, Y′, Z′).

22 $$\left[ {\begin{array}{ll} {X_{1}^{\prime} } \\ {Y_{1}^{\prime} } \\ {Z_{1}^{\prime} } \\ \end{array} } \right] = \left[ {\begin{array}{*{20}c} {\cos \theta } & 0 & {\sin \theta } \\ 0 & 1 & 0 \\ { - \sin \theta } & 0 & {\cos \theta } \\ \end{array} } \right]\left[ {\begin{array}{*{20}c} {X_{1} } \\ {Y_{1} } \\ {Z_{1} } \\ \end{array} } \right]$$

23 $$\left[ {\begin{array}{ll} {B_{X1}^{\prime} } \\ {B_{Y1}^{\prime} } \\ {B_{Z1}^{\prime} } \\ \end{array} } \right] = \left[ {\begin{array}{*{20}c} {\cos \theta } & 0 & { - \sin \theta } \\ 0 & 1 & 0 \\ {\sin \theta } & 0 & {\cos \theta } \\ \end{array} } \right]\left[ {\begin{array}{*{20}c} {B_{X1} } \\ {B_{Y1} } \\ {B_{Z1} } \\ \end{array} } \right]$$

If $P_{1}^{\prime} (X_{1}^{\prime} ,Y_{1}^{\prime} ,Z_{1}^{\prime} )$ is the new centre of the solenoid then $B_{X1}^{\prime} = B_{Y1}^{\prime} = 0.$

**Figure 11:**
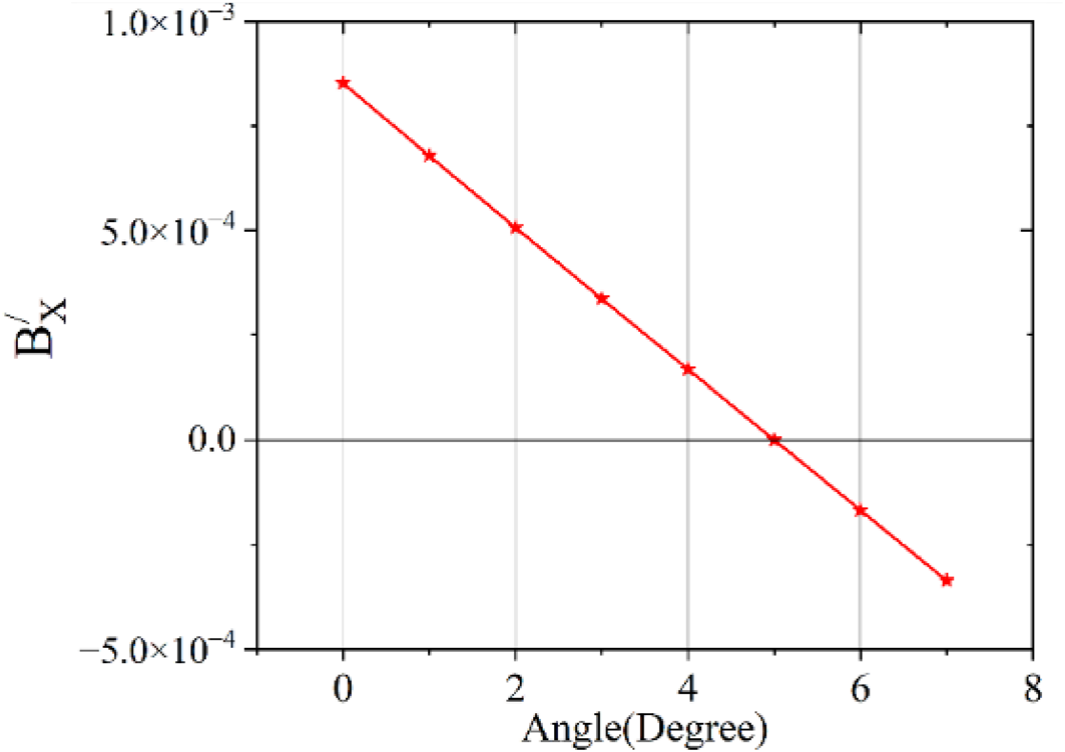
Variation $B_{X1}^{\prime} (T)$ with angle in degree.

To find the value of $\theta$_,_ a curve is drawn between $B_{X1}^{\prime} {\text{ and }}\theta$ using data given in Table 2 and shown in Fig. 11. The dimensions of the solenoid considered for the present study are as follows: Length, inner diameter and outer diameter are 0.4, 0.187, and 0.305 m, respectively. $B_{X1}^{\prime}$ is zero for angle 5 degree as obtained from Fig. 11. As we are detecting zero crossing, closer data points are not needed.

**Table 2:** Variation of $B_{X1}^{\prime} (T)$ with angle, $\theta$ at (0,0,0.2) in PB frame.

$\theta$	$X_{1}^{\prime} (m)$	$Z_{1}^{\prime} (m)$	$B_{X1} (T)$	$B_{Z1} (T)$	$B_{X1}^{\prime} (T)$ calculated
	Calculated		Simulated/measured value		
0	0.00E+00	2.00E−01	8.5335E−04	6.3154E−02	8.5335E−04
1	3.49E−03	2.00E−01	1.7770E−03	6.2921E−02	6.7864E−04
2	6.98E−03	2.00E−01	2.6973E−03	6.2723E−02	5.0668E−04
3	1.05E−02	2.00E−01	3.6158E−03	6.2559E−02	3.3671E−04
4	1.40E−02	2.00E−01	4.5339E−03	6.2429E−02	1.6804E−04
5	1.74E−02	1.99E−01	5.4534E−03	6.2333E−02	0.0000E+00
6	2.09E−02	1.99E−01	6.3757E−03	6.2268E−02	− 1.6804E−04
7	2.44E−02	1.99E−01	7.3025E−03	6.2237E−02	− 3.3671E−04

**Figure 12:**
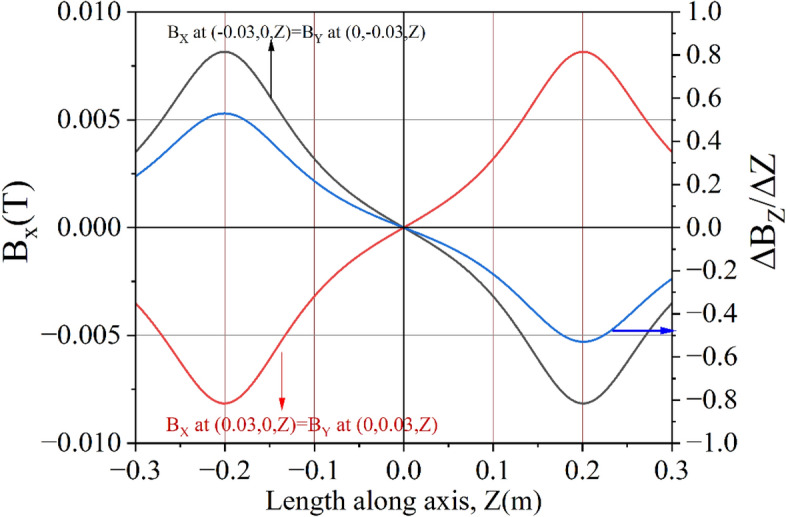
Variation of B_X_ (B_Y_) and derivative $\left( {\frac{{\partial B_{Z} }}{\partial Z}} \right)$ along the solenoid axis for different X(Y) as obtained from simulation after transforming the PB frame to the solenoid frame. This is exactly the same as expected from an ideal solenoid.

For $\theta$ = 5 degree, the point (− 0.03, 0, 0.2) in the PB frame will transform to (− 0.012, 0, 0.202) in the solenoid frame, and the corresponding field $\left( {B_{X1}^{\prime} ,B_{Y1}^{\prime} ,B_{Z1}^{\prime} } \right)$ will be (− 8.154E−03, 0, 6.259E−02). Similarly, the point (0.03, 0, 0.2) will transform to (0.047, 0, 0.197) and corresponding field $\left( {B_{X1}^{\prime} ,B_{Y1}^{\prime} ,B_{Z1}^{\prime} } \right)$ will be (8.154E−03, 0, 6.259E−02). Similarly, for the point (0, ± 0.03, 0.2), the corresponding field $\left( {B_{X1}^{\prime} ,B_{Y1}^{\prime} ,B_{Z1}^{\prime} } \right)$ will be (0, ± 8.154E−03, 6.259E−02). Therefore, the field is symmetric but opposite in sign, as in case of an ideal solenoid. The axis of the solenoid can, therefore, be determined efficiently and accurately by measuring magnetic field at a few points in the PB frame. Now, the reference frame of the PB is tilted by 5 degrees to match with the solenoid frame, and the simulated values of the fields are plotted in Fig. 12.

**Figure 13:**
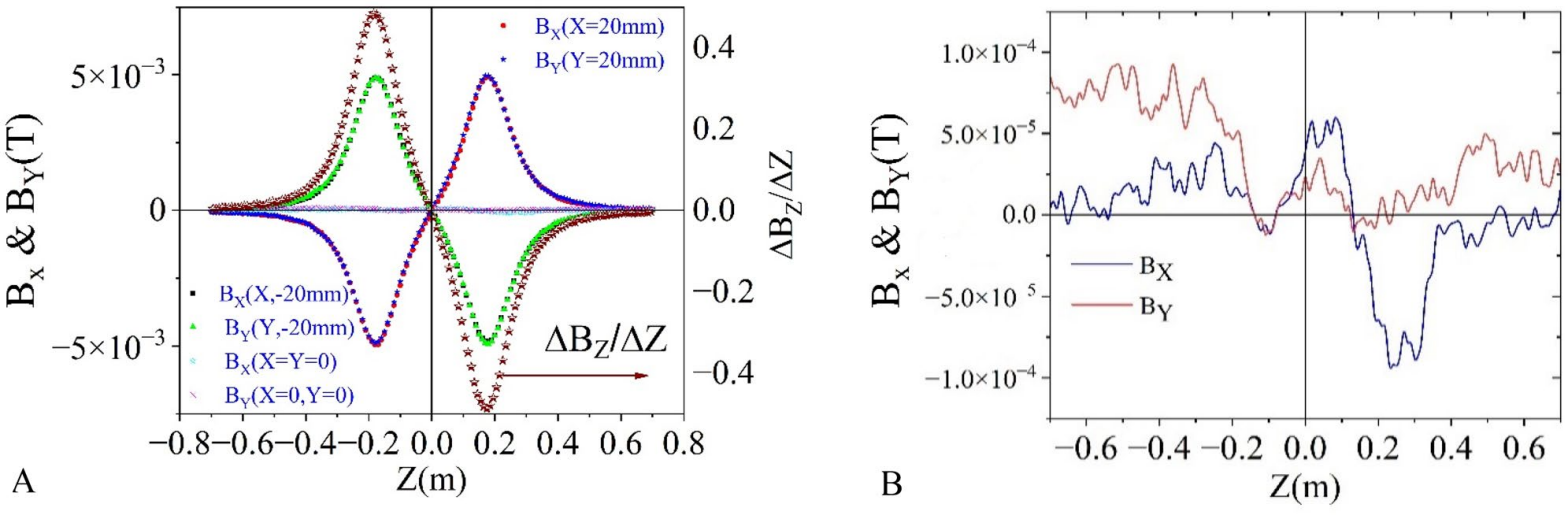
(**A**) Measured magnetic field data of B_X_ and B_Y_, and the derivative of the axial field along the axis of the solenoid. Field profiles are very close to the ideal solenoids as expected. Only at X = Y = 0, B_X_ and B_Y_ varies with Z within ± 1G rather than zero as in case of ideal solenoid (**B**).

However, in any practical magnet, this ideal nature may not be observed due to the presence of various unavoidable geometrical errors. Figure 13 illustrates the field variation of B_X_ and B_Y_ along the axis. For X = Y = 0, B_X_ and B_Y_ vary within ± 1 G rather than zero, as in case of an ideal solenoid. However, this error is very small and within an acceptable limit.

An efficient determination of accurate magnetic axis is not possible without using an integrated 3-axis sensor. A set of horizontal and vertical steering coils is placed inside each solenoid for beam steering, for use when required. This correction coils are capable of generating a 180 G-cm integrated field in each direction^27,28^. The derivative of the axial field is also observed to cross zero, as predicted by the theory. In our earlier machines, air core solenoids using OFHC Cu are used, but now proposed to use Al-strip solenoid with iron jacket because of several advantages and reduced cost. The benefits of using Al-strip solenoid with an iron jacket over OFHC-Cu are summarized in Table 3.

**Table 3:** Pros and cons of two different types of solenoids.

Comparison	Al-strip with iron jacket	OFHC-Cu (air core)
Availability	Country (India)	Imported
Cooling	Indirect cooling, Ordinary water	LCW water for cooling
Relative cost	0.5	1.0
Power loss at 1500 G	1.35 kW	5.15 kW
Inter turn insulation	Not required, Al_2_O_3_ oxide coated	Required
Fringe field near gun	< 2 G	< 20 G, additional shielding required
Overall volume	Relatively large	Compact

## Conclusions

We have conducted a thorough examination of charged particle dynamics within a solenoid, utilizing the conservation of canonical angular momentum, which can take on positive, negative, and zero values. The solutions have been rigorously justified through particle trajectories. Additionally, the analytical expression for the focal length of a solenoid is derived, along with the condition necessary to generate a parallel beam using a solenoid. The theoretical predictions have been verified through particle trajectories. Our findings reveals that the rotation angle of the charged particles inside a solenoid is solely depended on the $\int {B_{Z} \partial Z}$ that depends on the product of current in each turn and numbers of turns. It is independent of the geometrical parameters of the solenoid. Furthermore, we have introduced and experimentally demonstrated a method to tune the spherical aberration coefficient and the focal length of a solenoid by varying the inner diameter of the demountable iron disk connected at both ends. Particle trajectory observations indicate that not only the value of $C_{s}$, but also the ratio of inner radius of the solenoid to the radius of the beam, plays a significant role in reducing the beam size.

We have also proposed and experimentally demonstrated an efficient approach for accurately determining the axis and the magnetic centre of a solenoid, leveraging a 3-axis Hall sensor and probe positioning bench. This procedure has been instrumental in characterizing several solenoids for various applications, including 10 kW 9.5 MeV LINACs^28^. The accurate determination of the axis of a solenoid is crucial for optimizing the performance of charged particle dynamics, considering the axisymmetric magnetic field in all calculations.

Through this methodology, we have successfully designed, characterized, and utilized several solenoids for various projects. We have developed two types of solenoids (i) LCW cooled air core OFHC Cu and (ii) Anodized Al-strip with iron jacket employing indirect cooling with ordinary water. Pros and cons of the two different types of solenoids are presented. We proposed based on our analysis that Al-strip solenoids with iron jacket is cost effective and well-suited for a 9.5 MeV 10 kW LINAC project.

## Data Availability

The datasets used and/or analyzed during the current study available from the corresponding author on reasonable request.
